# Supplementary material for: Influenza A Virus Infection Reactivates Human Endogenous Retroviruses Associated with Modulation of Antiviral Immunity
Source: Viruses. 2022 Jul 21;14(7):1591. doi: 10.3390/v14071591 (PMC9320126; doi:10.3390/v14071591)
Supplement: Supplementary file 1 [file viruses-14-01591-s001.zip › MotifAnalysis.zip!/MotifOutput_PR8M/homerResults/motif15.similar.html]

motif15

## Information for motif15

A
G
T
C
A
C
G
T
A
C
T
G
A
C
T
G
A
C
T
G
A
G
T
C
A
C
G
T
A
G
T
C
A
G
T
C
A
C
G
T
A
C
T
G
C
G
T
A
  
Reverse Opposite:  

A
C
G
T
A
G
T
C
C
G
T
A
A
C
T
G
A
C
T
G
C
G
T
A
A
C
T
G
A
G
T
C
A
G
T
C
A
G
T
C
C
G
T
A
A
C
T
G
  

|  |  |
| --- | --- |
| p-value: | 1e-12 |
| log p-value: | -2.826e+01 |
| Information Content per bp: | 1.530 |
| Number of Target Sequences with motif | 5.0 |
| Percentage of Target Sequences with motif | 21.74% |
| Number of Background Sequences with motif | 21.0 |
| Percentage of Background Sequences with motif | 0.05% |
| Average Position of motif in Targets | 24.2 +/- 14.5bp |
| Average Position of motif in Background | 99.4 +/- 118.4bp |
| Strand Bias (log2 ratio + to - strand density) | 10.0 |
| Multiplicity (# of sites on avg that occur together) | 1.00 |
| Motif File: | file (matrix) reverse opposite |

### Similar de novo motifs found

|  |  |  |  |  |  |  |  |
| --- | --- | --- | --- | --- | --- | --- | --- |
| Rank | Match Score | Redundant Motif | P-value | log P-value | % of Targets | % of Background | Motif file |
